# Correlation between the drug concentration of polymyxin B and polymyxin B‐associated acute kidney injury in critically ill patients: A prospective study

**DOI:** 10.1002/prp2.1010

**Published:** 2022-10-07

**Authors:** Ying Xu, Pei Liang, Ning Liu, Danjiang Dong, Qin Gu, Xinying Wang

**Affiliations:** ^1^ Surgical Intensive Care Unit (SICU), Department of General Surgery Jinling Hospital of Nanjing Medical University Nanjing China; ^2^ Intensive Care Unit Drum Tower Hospital Affiliated to Nanjing University School of Medicine Nanjing Jiangsu China; ^3^ Department of Pharmacy Drum Tower Hospital Affiliated to Nanjing University School of Medicine Nanjing China

**Keywords:** acute kidney injury, area under the concentration–time curve, critically ill patients, drug concentration, polymyxin

## Abstract

In recent years, polymyxin B‐associated acute kidney injury (PB‐AKI) in critically ill patients has been reported frequently, but polymyxin B (PB) is mainly cleared through non‐renal pathways, and the reasons of PB‐AKI remain unclear. The aim of this study was to investigate the relationship between the serum concentration of PB and PB‐AKI. We conducted a prospective cohort study in an intensive care unit between May 2019 and July 2021. Over the study period, 52 patients were included and divided into an AKI group (*n* = 26) and a non‐AKI group (*n* = 26). The loading dose of PB in the AKI group was significantly higher than that in the non‐AKI group. The *C*_1/2_, *C*_min_, and estimated area under the concentration–time curve (AUC)_0–24_ of PB in the AKI group were dramatically increased compared with those in the non‐AKI group, but the *C*_max_ between the two groups showed no differences. Upon obtaining the ROC curve, the areas for the *C*_1/2_, *C*_min_, and estimated AUC_0–24_ were 0.742, 0.710, and 0.710, respectively. The sensitivity was ascertained to be 61.54%, and the specificity was 76.92% when the cutoff value for the estimated AUC_0–24_ of 97.72 mg·h/L was used preferentially. The incidence of PB‐AKI is high and related to the loading dose of PB. PB‐AKI could be predicted when the estimated AUC_0–24_ of PB was greater than 97.72 mg·h/L.

**Abbreviations**
AKI: acute kidney injury
ALT: alanine aminotransferase
APACHE II: Acute Physiology and Chronic Health Evaluation
AST: aspartate transaminase
AUC: the concentration–time curve over a 24‐h period
C1/2: a middle serum concentration through a dosing interval
Ccr: creatinine clearance
*C*_max_
: peak serum concentration of PB
Cmin: trough serum concentration of PB
CPR: cardiopulmonary resuscitation
CRP: C‐reactive protein
Css: steady‐state plasma concentration
PB: polymyxin B
Scr: serum creatinine
WBC: white blood cell

## 1 INTRODUCTION

Polymyxin is a kind of non‐ribosomal basic peptide antibacterial drug that plays a rapid bactericidal role mainly by destroying the integrity of the cell membrane. It was used in the clinical treatment of anti‐gram‐negative bacterial infections as early as the 1950s and gradually replaced due to its narrow antibacterial spectrum and obvious neurotoxicity and renal toxicity. Currently, with the increasing infection rate of multidrug‐resistant gram‐negative bacteria worldwide, polymyxin is once again regarded as the last line of defense against gram‐negative bacterial infection.^1^

According to their chemical structure, polymyxin can be divided into five different families: polymyxin A, B, C, D, and E.^2^ Only two are clinically used: polymyxin B (PB) and polymyxin E. PB is a mixture of more than 30 polymyxin compounds (mainly PB1, PB2, PB3, and PB4). The most important components are B1 (PB1), which contains aliphatic (S)‐6‐methyl‐octyl, and polymyxin B2 (PB2), which contains aliphatic 6‐methyl‐heptyl.^3^ At present, PB1 and PB2 are mainly used in pharmacokinetic (PK) determination, accounting for approximately 85% of PB use.^4^ Compared with polymyxin E, PB has an excellent PK effect and lower renal toxicity and is more frequently administered in the clinical practice. According to the drug instructions, the commonly used dose of PB is 1.5–2.5 mg/kg per day, divided evenly into two doses. PB was mainly cleared by non‐renal pathway and only 4% of PB is cleared from the kidney, but 90%–95% of PB is reabsorbed from kidney tubules.^5^, ^6^ The non‐renal pathway of PB to date is not clear, but all four components of PB have been detected in bile without undergoing a metabolic process.^7^ Therefore, based on the consideration of efficacy, the latest consensus guidelines for PB from the Infectious Diseases Society of America do not suggest adjusting the dose according to the creatinine clearance (Ccr) rate.^8^

However, PB‐associated acute kidney injury (PB‐AKI) has been increasingly reported in recent years, with an incidence ranging from 12.7% to 60% according to different diagnostic criteria.^9^, ^10^, ^11^, ^12^, ^13^ Some researchers have suggested that the risk factors for PB‐AKI are related not only to old age, shock, and basic renal function, but also to nephrotoxic drugs (such as vancomycin, aminoglycosides, contrast agents, and nonsteroidal anti‐inflammatory drugs).^14^, ^15^, ^16^ Studies have also shown that the total dose, load dose, or maintenance dose ≥150 mg/d of PB are independent risk factors for PB‐AKI,^13^, ^17^, ^18^ but whether they are related to the pharmacokinetics (PK) of PB is not clear. To date, clinical data on the association between PK and PB‐AKI are limited.^19^ Moreover, the factors of AKI in critically ill patients are complex, and incidence is high and may occur more frequently after PB administration. To date, limited literature has been found on the correlation between the PK of PB and the occurrence of AKI in critically ill patients.

On the above basis, this study reviewed the PK of PB and renal function changes in critically ill patients without basic renal insufficiency after PB administration and explored the relationship between PK and PB‐AKI.

## 2 MATERIALS AND METHODS

### 2.1 Study design and patients

A prospective cohort study was conducted in the 45‐bed adult ICU of Drum Tower Hospital affiliated with the Medical School of Nanjing University (Nanjing) between 1 May 2019 and 30 July 2021. This study was approved by the Ethics Committee of Drum Tower Hospital affiliated with the Medical School of Nanjing University, and written informed consent was obtained from all patients or responsible caregivers.

Inclusion criteria included age ≥ 18 years and intravenous administration of PB for more than 3 days. Exclusion criteria included PB administration of a daily dose of 1.5–2.5 mg/kg <3 days, underlying chronic renal insufficiency or existing AKI, or renal replacement therapy already performed. The loading dose and maintenance dose of PB for each patient depended on the doctors' decisions at a daily dose of 1.5–2.5 mg/kg in evenly two doses. PB was administered as an intermittent infusion over approximately 1 h. Patients were divided into an AKI group and a non‐AKI group according to whether they developed new AKI after PB application according to Kidney Disease Improving Global Outcomes (KDIGO) diagnostic criteria.^20^ An increase in serum creatinine (Scr) to 50% of the base value within 7 days or an increase in Scr of more than 26.5 μmol/L within 2 days or oliguria is diagnosed as AKI according to KDIGO's diagnostic criteria.

### 2.2 Data collection

The data collection was performed by trained staff and the data were entered into a case report form. The demographic and clinical data for the patients, which included sex, age, weight, Acute Physiology and Chronic Health Evaluation II (APACHE II) score, underlying diseases, and comorbidities, were recorded. Laboratory parameters, including blood cell counts, procalcitonin, C‐reactive protein (CRP), alanine aminotransferase (ALT), aspartate transaminase (AST), total bilirubin, blood urea nitrogen, Scr, and Ccr, were monitored periodically. In addition, information on PB dosing (loading dose, daily dose, total dose and frequency, and duration days) and concomitant use of other antibiotics was collected.

### 2.3 PK sample collection and analysis

**FIGURE 1:**
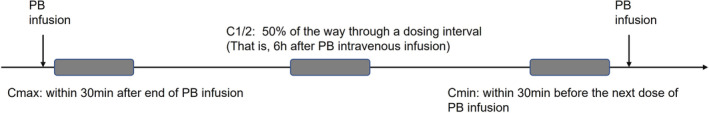
Blood sampling scheme of PB plasma concentration. *C*_1/2_, the serum concentration in the middle of a dosing interval; *C*_max_, peak serum concentration of PB; *C*_min_, trough serum concentration of PB; PB, polymyxin B

The estimated area under the plasma concentration–time curve from 0 to 24 h (AUC_0–24_) was the main PK variable for PB. From each patient, three blood samples were taken after at least six doses of PB: blood sample A (*C*_max_) was collected within 30 min after the end of intravenous infusion of PB for the peak concentration, blood sample B (*C*_1/2_) was a mid‐dose blood sample taken 50% of the way through a dosing interval, and blood sample C (*C*_min_) was a pre‐dose concentration taken at the end of a dosing interval (within 30 min of the next dose, see Figure 1). The serum was separated and stored at −70°C until assay.

The PB1 and PB2 serum concentrations were measured using a validated ultrahigh performance liquid chromatography–tandem mass spectrometry assay method.^21^ The *C*_max_, *C*_1/2_, and *C*_min_ of steady‐state (after at least 5 doses) PB1 and PB2 components were determined according to the above method:

$$C_{\max}=C_{\max-\mathrm{PB}1}+C_{\max-\mathrm{PB}2},$$

$$C_{1/2}=C_{1/2-\mathrm{PB}1}+C_{1/2-\mathrm{PB}2},$$

$$C_{\min}=C_{\min-\mathrm{PB}1}+C_{\min-\mathrm{PB}2},$$

The estimated area under the concentration–time curve from 0 to 12 h (AUC_0–12_) was calculated using the linear trapezoidal approximation^22^ and the estimated AUC_0–24_ from 0 to 24 h (AUC_0–24_) was calculated as double the AUC_0–12,_ ^23^ assuming a steady state (after at least 4–5 doses) when PB was administered every 12 h.

### 2.4 Statistical analyses

Data are reported as categorical variables or numerical variables. Continuous normally distributed numerical variables are expressed as the means ± standard deviations, and continuous non‐normally distributed variables are expressed as the median (interquartile range). ANOVA test was used to compare the continuous normally distributed variables, Kruskal–Wallis rank sum test was used to compare the continuous non‐normally distributed variables, and Chi‐square test was used to compare the rates. The sensitivity and specificity of *C*_1/2_, *C*_min_, and estimated AUC_0–24_ for the development of AKI were determined using ROC curve analysis. AKI occurrence as the outcome, to maximize the Jorden index to find the best entry point. Statistical significance was defined as *p* < .05. SPSS version 22.0 (SPSS Inc.) was used for all statistical calculations.

## 3 RESULTS

### 3.1 Demographics and clinical characteristics in the overall population

**FIGURE 2:**
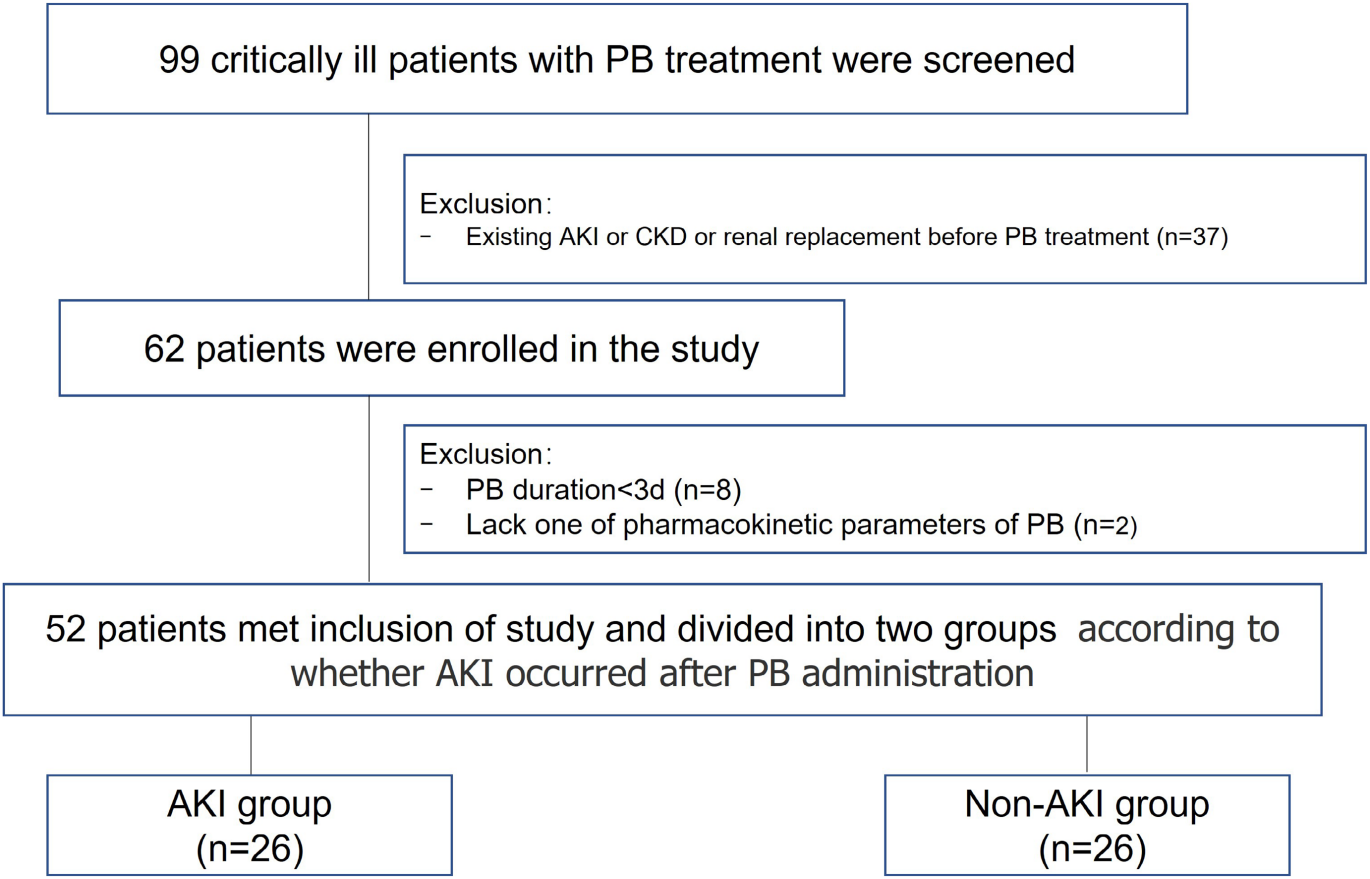
Schematic flow chart of patient enrolment. AKI, acute kidney injury; PB, polymyxin B

During the study period, 99 critically ill patients who received PB treatment were screened. Of these, 37 patients had already had AKI or chronic kidney disease or had undergone renal replacement therapy before PB administration, and 62 patients met the inclusion criteria and were considered for the prospective analysis. Among them, eight patients had PB administration for less than 3 days, and two patients lacked at least one PB serum concentration measurement. Finally, a total of 52 patients were included in the study and divided into AKI (*n* = 26) and non‐AKI (*n* = 26) groups according to whether AKI occurred after PB administration (Figure 2).

The overall characteristics of the two groups are shown in Table 1. The proportion of males in the AKI group was 80.8%, which was significantly higher than 50% in the non‐AKI group (*p* < .05). The APACHE II score in the AKI group was obviously higher than that in the non‐AKI group (18.46 vs. 11.32, *p* < .05). The proportions of respiratory failure, shock, and bacteremia in the AKI group were significantly higher than those in the non‐AKI group (all *p* < .05).

**TABLE 1:** Overall characteristics of the AKI and non‐AKI groups

	AKI group (*n* = 26)	Non‐AKI group (*n* = 26)	*p* value
Demographics			
Age (years)	64.73 ± 16.48	61.08 ± 14.96	.407
Sex (male), *n* (%)	21 (80.8)	13 (50)	.020
APACHE II score	18.46 ± 4.34	11.32 ± 5.19	<.001
Comorbidities (*n*,%)			
Hypertension	8 (30.8)	13 (50)	.129
Diabetes	3 (11.5)	6 (23.1)	.233
Respiratory failure	16 (61.5)	7 (26.9)	.012
Shock	14 (53.8)	3 (11.5)	.001
Bacteremia	17 (65.4)	6 (23.1)	.002
Malignant disease	6 (23.1)	1 (3.8)	.05
Immune system diseases	17 (19.5)	20 (19.8)	.556
Laboratory findings (before polymyxin B treatment) (median, IQR)			
WBC (*10^9^/L)	10.5 (4.75–18)	9 (7.45–12)	.830
Platelets (*10^9^/L)	181 (78–315)	212 (201–358)	.093
PCT (mg/L)	0.1 (0.01–1.1)	0.234 (0.01–1)	.784
CRP (mg/L)	91 (79–110)	15 (3–84)	.011
ALT (U/L)	20 (12–36)	34 (32.5–53.5)	.003
AST (U/L)	22.5 (16.75–42.25)	48 (28–52)	.012
TB (μmol/L)	4.5 (3–11.25)	3 (2–15.5)	.430
BUN (mmol/L)	10.82 (7–16)	9 (6–10)	.138
Scr (μmol/L)	54.5 (43–68.25)	41 (32–59)	.026
Ccr (mL/min)	102.37 (59.05–110.2)	89 (60.44–136.12)	.728
Outcomes			
LOS	29.54 ± 12.62	34.9 ± 12.36	.320
28‐d mortality (*n*, %)	12 (46.2)	5 (22.7)	.082

Abbreviations: AKI, acute kidney injury; ALT, alanine aminotransferase; APACHE II, Acute Physiology and Chronic Health Evaluation; AST, aspartate transaminase; BUN, blood urea nitrogen; Ccr, creatinine clearance; CPR, cardiopulmonary resuscitation; CRP, C‐reactive protein; LOS, length of stay; PCT, procalcitonin; Scr; serum creatinine; TB, total bilirubin; WBC; white blood cell.

The laboratory findings and prognoses of patients in the two groups before PB administration are also shown in Table 1. Serum CRP in the AKI group was six times higher than that in the non‐AKI group and serum Scr levels in the AKI group were slightly increased compared to the non‐AKI group, while ALT and AST levels in the non‐AKI group were higher than those in the AKI group (all *p* < .05). There was no significant difference in the length of hospital stay between the two groups, but there was an increase in 28‐d mortality in the AKI group compared to the non‐AKI group, although there was no significant difference (*p* = .082).

PB dosage, duration, and combined drugs in the two groups are shown in Table 2. The median loading doses of PB in the two groups were 124.04 mg and 102.89 mg, respectively, with obvious significant differences (*p* < .05), while there were no differences in the PB daily dose, daily dose/body weight, duration, or total dose of treatment between the two groups (*p* > .05). The proportion of patients in the non‐AKI group who received PB combined with tigecycline was significantly higher than that in the AKI group (*p* < .05), but there was no significant difference in the proportion of patients who received carbapenems and β‐lactam antibiotics between the two groups (*p* > .05).

**TABLE 2:** Comparison of treatment with polymyxin B in AKI and non‐AKI groups

	AKI group (*n* = 26)	Non‐AKI group (*n* = 26)	*p* value
Loading dosage (mg)	124.04 ± 27.8	102.89 ± 32.66	.015
Daily dosage (mg/d)	131.92 ± 25.62	126.92 ± 25.42	.483
Daily dosage/weight(mg/kg/d)	2.20 ± 0.40	2.12 ± 0.34	.428
Duration(days)	12.85 ± 5.95	10.75 ± 4.36	.165
Total dose (mg)	1789.81 ± 779.58	1503.13 ± 707.16	.181
Concomitant with β‐lactam, non‐carbapenems (*n*,%)	4 (15.4)	8 (30.8)	.162
Concomitant with carbapenem, (*n*,%)	8 (30.8)	7 (26.9)	.500
Concomitant with tigecycline, (*n*,%)	26 (29.9)	43 (42.6)	.049

Abbreviation: AKI, acute kidney injury.

### 3.2 The PK parameters in the AKI and non‐AKI groups

As shown in Table 3, the median value of *C*_max_, *C*_1/2_, *C*_min_, and estimated AUC_0–24_ of AKI group were 6.80, 3.94, 2.72 mg/L, and 102.37 mg·h/L, while 6.65, 2.77, 1.66, and 85.5 mg·h/L, respectively, in the non‐AKI group. There were no significant differences in *C*_max_ between the two groups (*p* > .05), but *C*_1/2_ and *C*_min_ in the AKI group were obviously increased compared with those in the non‐AKI group (*p* < .05), and the estimated AUC_0–24_ in the AKI group was also higher than that in the non‐AKI group (*p* < .05).

**TABLE 3:** PK/PD parameters of PB in AKI and non‐AKI groups

PK/PD parameters (median, IQR)	Total patients (*N* = 52)	AKI group (*n* = 26)	Non‐AKI group (*n* = 26)	*p* value
*C*_max_ (mg/L)	6.69 (5.56–8.36)	6.80 (6.40–8.37)	6.65 (5.14–8.24)	.390
*C*_1/2_ (mg/L)	3.25 (2.574.22)	3.94 (2.99–4.71)	2.77 (2.29–3.42)	.003
*C*_min_ (mg/L)	2.08 (1.39–2.90)	2.72 (1.96–3.01)	1.66 (1.32–2.38)	.009
Estimated AUC_0–24_ (mg·h/L)	92.07 (77.11–118.02)	102.37 (86.18–120.61)	85.5 (69.16–97.55)	.009

Abbreviations: AKI, acute kidney injury; AUC_0–24_, the concentration–time curve over a 24‐h period; *C*_1/2_, a middle serum concentration through a dosing interval; *C*_max_, peak serum concentration of PB; *C*_min_, trough serum concentration of PB; PB, polymyxin B.

### 3.3 ROC curves of PK parameters of PB for predicting the occurrence of AKI in critically ill patients

**FIGURE 3:**
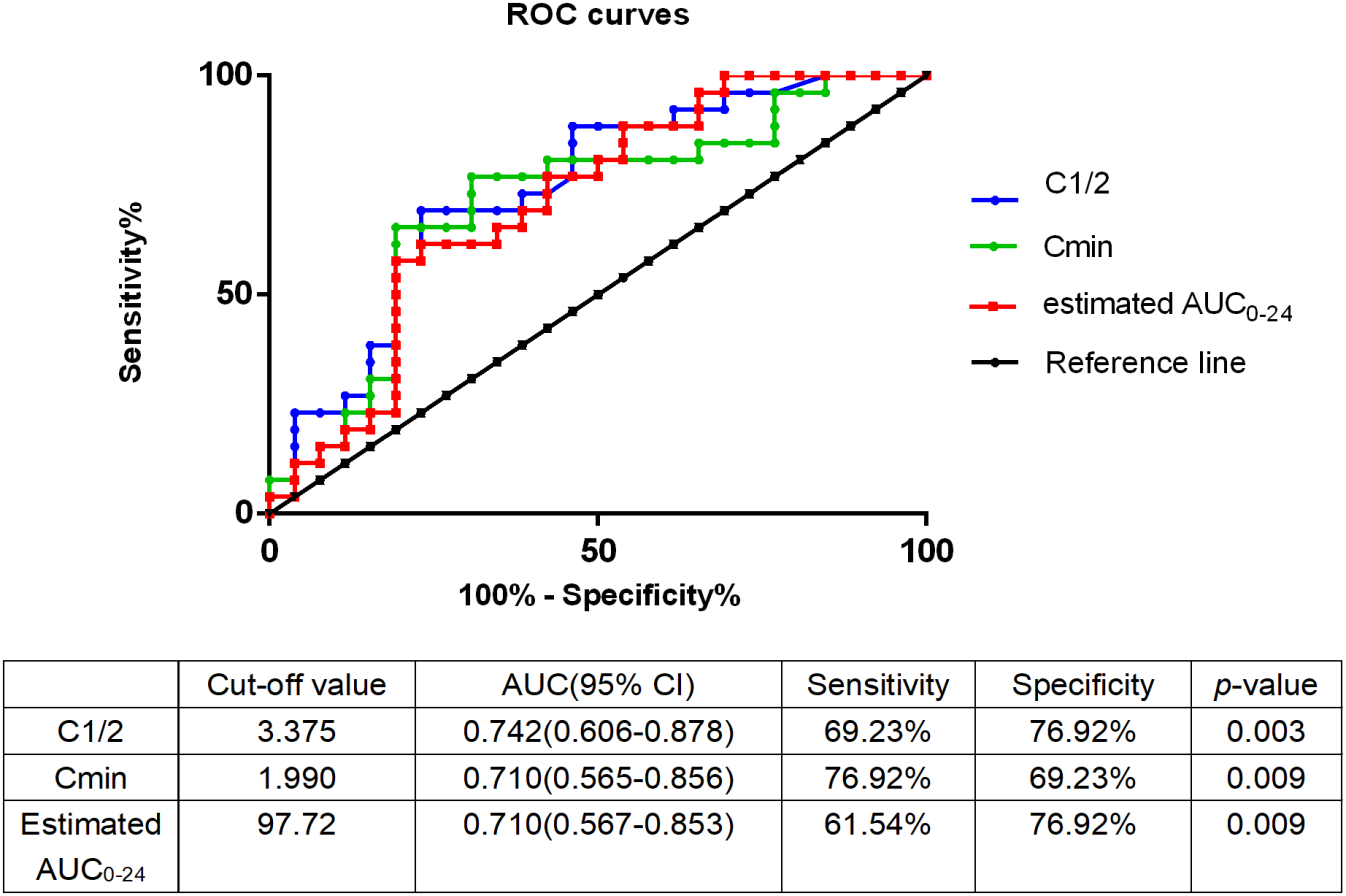
ROC curves for the sensitivity and specificity of PK levels for the development of PB‐AKI. AKI, acute kidney injury; AUC_0–24_, the concentration‐time curve over a 24‐h period; *C*_1/2_, the serum concentration in the middle of a dosing interval; *C*_min_, trough serum concentration of PB; PB, polymyxin B

The sensitivity and specificity of PK parameters for predicting the occurrence of PB‐AKI were assessed according to the ROC curve, as shown in Figure 3. Upon obtaining the ROC curve, the areas for *C*_1/2_, *C*_min_, and estimated AUC_0–24_ under the ROC curves were 0.742, 0.710, and 0.710, respectively. The sensitivity was ascertained to be 69.23%, and the specificity was 76.92% when the cutoff value for *C*_1/2_ of 3.375 mg/L was used preferentially (*p* = .003). However, to balance drug efficacy and safety simultaneously, estimated AUC_0–24_ can be used to predict the occurrence of PB‐AKI, with a sensitivity and specificity of 61.54% and 76.92%, respectively, and a cutoff value of 97.72 mg·h/L. The consistency between the predicting PB‐AKI based on estimated AUC_0–24_ ≥ 97.72 mg·h/L and actual PB‐AKI was shown in Table 4.

**TABLE 4:** Consistency between predicting PB‐AKI based on estimated AUC_0–24_ ≥ 97.72 mg·h/L and actual PB‐AKI

	Actual PB‐AKI	
	Positive	Negative
Predicting		
Positive	16	6
PB‐AKI		
Negative	10	20

Abbreviations: AKI, acute kidney injury; AUC_0–24_, the concentration–time curve over a 24‐h period; PB, polymyxin B.

## 4 DISCUSSION

This study suggested that the incidence of AKI caused by intravenous PB was as high as 50% (26/52) in critically ill patients. Estimated AUC_0–24_ is a predictor for PB‐AKI, and an estimated AUC_0–24_ ≥ 97.72 mg·h/L was identified as a significant breakpoint for PB‐AKI. This is consistent with previous clinical studies that also found that an estimated AUC_0–24_ > 100 mg·h/L was a good predictor for the probability of nephrotoxicity of PB treatment.^19^

Common adverse reactions associated with polymyxin include nephrotoxicity and neurotoxicity, of which nephrotoxicity accounts for the majority of adverse reactions.^24^, ^25^ The drug elimination mechanisms involved in PB include renal and nonrenal pathways. PB can be reabsorbed in renal tubules, with only approximately 4% excreted through the kidney.^5^ Although the nonrenal mechanism of PB clearance is not fully elucidated, it is considered to be the main pathway of PB clearance.^26^ Therefore, the Ccr rate has little influence on the metabolism of PB, and there is no need to adjust the dosage for patients with renal insufficiency.^27^

Although clearance of PB is mainly through nonrenal excretion, up to 90%–95% of PB filters through and is reabsorbed by renal tubular cells.^6^ In recent years, PB‐AKI has been increasingly reported, which is a problem that should not be ignored.^13^, ^17^, ^28^ Studies have shown that the main features of PB‐AKI include acute renal tubular injury and reduced Ccr.^25^ In a mouse study, PB distribution in renal tissue after intravenous administration was detected using PB‐specific monoclonal antibody immunostaining and was mainly concentrated in the renal cortex, especially in the proximal tubular cells.^29^ Mohammad et al. also reported that the concentration of polymyxin in human renal tubular cells was 4760 times higher than that in extracellular cells, indicating an abnormal aggregation of polymyxin in these cells.^30^ Recently, studies have confirmed that megalin is a key endocytic receptor for reabsorption of proteins and small bioactive molecules in glomerular filtrate and mediates significant reabsorption of polymyxin in renal tubular cells.^31^, ^32^ In addition, polymyxin has a competitive inhibitory effect on the binding of cytochrome C to megalin. In the megalin‐shed mouse study, the accumulation of polymyxin in the kidney was reduced, and the excretion of colistin in the urine increased, suggesting that megalin is important for the reabsorption of colistin by tubule cells.^32^

The occurrence of PB‐AKI may be related to the drug dose of PB. In this study, PB‐AKI was associated with the loading dose of PB but not with the daily dose, daily dose/weight, duration, or total dose of treatment, and recent clinical studies have come to the same conclusion.^33^ Other studies have shown that a daily dose of PB ≥ 150 mg is a risk factor for nephrotoxicity.^18^ Soares et al. also found that a cumulative dose of PB > 1000 mg in critically ill patients was an independent risk factor for AKI.^34^ Different studies have shown different results, but in general, the occurrence of PB‐AKI is dose dependent, whether load dose, daily dose, or total dose. All doses above a certain level have been confirmed to be related to the occurrence of PB‐AKI in different studies, which may be related to the increased local concentration of renal tubular cells caused by the reabsorption of PB in renal tubules. An animal experiment showed that the pathogenesis of PB‐AKI involves drug reabsorption and deposition in renal proximal convoluted tubule cells, which then induces oxidative stress and cell cycle arrest and apoptosis, and this cytotoxic damage is dose dependent.^35^

The occurrence of PB‐AKI may also be related to the PK of PB. According to the international consensus on polymyxin jointly released by several international academic organizations or institutions in 2019,^8^ the efficacy and toxicity consideration of PB can refer to the target range of the estimated AUC_0–24,_ which should be within 50–100 mg·h/L. However, the estimated AUC_0–24_ of the AKI group was significantly higher than that of the non‐AKI group, and the ROC curve also confirmed that an estimated AUC_0–24_ ≥ 97.72 mg·h/L could predict the occurrence of AKI. Peile Wang et al. similarly concluded that an estimated AUC_0–24_ greater than 100 mg·h/L of PB was associated with an increased risk of nephrotoxicity.^19^ Therefore, the increased concentration of PB may be an important reason for the occurrence of PB‐AKI, but the clinical data are limited at present.

Interestingly, in our study, male was more predominant in AKI group than in non‐AKI study and the reason was unclear. A study had shown that the combination of PB with selective estrogen receptor modulators can enhance the antibacterial activity of PB against XDR‐negative bacteria, which may be a reasonable explanation,^36^ but further studies are needed to confirm whether gender is associated with PB‐AKI.

This study had several limitations. First, this is a single‐center prospective cohort analysis with a relatively small number of patients. Due to the low compliance of PB concentration collection, the enrolment rate was affected and the sample size should be expanded in the future to further verify the reliability of the results. Second, the trapezoid method was used in this study to calculate the estimated AUC_0–24_, which was not the exact AUC_0–24_ of the patients, but this study shows that this method is suitable for clinical patients. Finally, we did not investigate the recovery of PB‐AKI after drug withdrawal.

In conclusion, AUC_0–24_ is associated with PB‐AKI, and an estimated AUC_0–24_ ≥ 97.72 mg·h/L can predict the occurrence of PB‐AKI. Therefore, to consider both efficacy and safety, it is best to monitor the drug concentration of PB and maintain an estimated AUC_0–24_ within 50–97.72 mg·h/L.

## AUTHOR CONTRIBUTIONS

All work has been approved by all co‐authors. YX and XYW made substantial contributions to the conception and design of the study; the data were acquired by YX, NL, DJD, PL, and QG; the analysis and interpretation of the data were performed by YX; YX and XYW wrote the draft of the article and revised it critically for intellectual content. The final version was approved by all authors.

## FUNDING INFORMATION

Financial funding support was received from the Medical Technology Development Project of Nanjing (Grant No. YKK15055).

## CONFLICT OF INTEREST

The authors declare that they have no competing interests.

## ETHICAL APPROVAL AND CONSENT TO PARTICIPATE

This study was approved by the Ethics Committee of Drum Tower Hospital affiliated with the Medical School of Nanjing University(ethical code: 2021‐522‐02). The need to obtain informed consent from individual patients was waived given the retrospective nature of the study. All methods were carried out in accordance with relevant guidelines and regulations.

## CONSENT FOR PUBLICATION

Not applicable.

## Data Availability

The datasets used and/or analyzed during the current study are available from the corresponding author on reasonable request.
